# Peroxin MoPex22 Regulates the Import of Peroxisomal Matrix Proteins and Appressorium-Mediated Plant Infection in *Magnaporthe oryzae*

**DOI:** 10.3390/jof10020143

**Published:** 2024-02-10

**Authors:** Rangrang Chen, Kailun Lu, Lina Yang, Jihong Jiang, Lianwei Li

**Affiliations:** 1 The Key Laboratory of Biotechnology for Medicinal Plant of Jiangsu Province, School of Life Sciences, Jiangsu Normal University, Xuzhou 221116, China; 2020190640@jsnu.edu.cn (R.C.); 1020220017@jsnu.edu.cn (K.L.); 2 College of Plant Protection, Yangzhou University, Yangzhou 225009, China; 007334@yzu.edu.cn

**Keywords:** *Magnaporthe oryzae*, appressorium, peroxisome, pathogenicity

## Abstract

*Magnaporthe oryzae*, the pathogen responsible for rice blast disease, utilizes specialized infection structures known as appressoria to breach the leaf cuticle and establish intracellular, infectious hyphae. Our study demonstrates that the peroxin MoPex22 is crucial for appressorium function, specifically for the development of primary penetration hyphae. The ∆*Mopex22* mutant exhibited slow growth, reduced aerial hyphae, and almost complete loss of virulence. Specifically, despite the mutant’s capability to form appressoria, it showed abnormalities during appressorium development, including reduced turgor, increased permeability of the appressorium wall, failure to form septin rings, and significantly decreased ability to penetrate host cells. Additionally, there was a delay in the degradation of lipid droplets during conidial germination and appressorium development. Consistent with these findings, the Δ*Mopex22* mutant showed an inefficient utilization of long-chain fatty acids and defects in cell wall integrity. Moreover, our findings indicate that MoPex22 acts as an anchor for MoPex4, facilitating the localization of MoPex4 to peroxisomes. Together with MoPex4, it affects the function of MoPex5, thus regulating the import of peroxisomal matrix proteins. Overall, these results highlight the essential role of MoPex22 in regulating the transport of peroxisomal matrix proteins, which affect fatty acid metabolism, glycerol accumulation, cell wall integrity, growth, appressorium development, and the pathogenicity of *M. oryzae*. This study provides valuable insights into the significance of peroxin functions in fungal biology and appressorium-mediated plant infection.

## 1. Introduction

Rice blast, caused by *M. oryzae*, occurs at various stages of rice growth and threatens global food security [1]. *M. oryzae* relies on a specialized structure known as the appressorium for infecting host rice [2]. Appressorium differentiation involves autophagy in the conidium, leading to programmed cell death and the mobilization of the contents from the three-celled spore to the infection cell. Efficient recycling of conidium contents is vital for the proper functioning of the appressorium [3,4]. The significant turgor pressure in appressorium is generated by the accumulation of glycerol, acting as a compatible solute, leading to water influx into the cell and subsequent hydrostatic pressure creation [5]. Melanin in the appressorium’s cell wall maintains glycerol by reducing its porosity [6]. Appressorium turgor is monitored by a sensor kinase, Sln1, and when a threshold is reached, septin GTPases in the appressorium pore form a hetero-oligomeric complex that serves as a scaffold for cortical F-actin at the appressorium’s base [7,8]. This process generates the necessary force to penetrate the cuticle using a rigid penetration hypha [8]. After entering the leaf, *M. oryzae* colonizes the initial epidermal cell, actively suppresses plant immunity, and spreads to adjacent cells [9,10]. Within a few days, disease lesions emerge, from which the fungus sporulates and colonizes neighboring plants.

Peroxisomes fulfill numerous crucial roles, such as the synthesis of ether phospholipids, the β-oxidation of long-chain fatty acids, the regulation of redox homeostasis, the utilization of carbon sources, and the production of melanin [11,12,13,14]. Proteins involved in peroxisomal biogenesis are referred to as peroxins, which are encoded by PEX genes. Since peroxisomes lack their own DNA and protein synthesis machinery, the membrane proteins and matrix proteins of peroxisomes are encoded by nuclear genes and synthesized in the cytosol [15]. Subsequently, they are post-translationally targeted to peroxisomes using the peroxisomal membrane targeting signal (PTS1 or PTS2) [15]. The import of peroxisomal matrix proteins is mediated by two receptors, Pex5 and Pex7, which recognize type I and type II peroxisomal targeting signals (PTSs), respectively [16,17,18,19,20,21]. The PTS1 receptor Pex5 has been shown to enter the peroxisomal matrix together with its cargo and to recycle back to the cytosol after dissociation from its cargo [22]. The recycling of PTS1 receptors to the cytosol is dependent on the interaction between Pex4, a ubiquitin-conjugating enzyme, and its membrane anchor, Pex22 [23,24]. In *Saccharomyces cerevisiae*, the C-terminus of Pex22 binds to Pex4, anchoring it to the peroxisome membrane to facilitate the appropriate ubiquitination of the PTS1 receptor. This ubiquitination is crucial for the release of the PTS1 receptor into the cytoplasmic matrix [25,26]. Consequently, the release of the receptor directly affects the transport of matrix proteins and the synthesis of peroxisomes [27].

The role of peroxins in the pathogenicity of various plant-pathogenic fungal species has been established in recent years. For instance, in *Fusarium graminearum*, several peroxins, including FgPex1, FgPex2, FgPex4, FgPex5, FgPex6, FgPex7, FgPex10, FgPex12, FgPex13, FgPex14, and FgPex33, have been shown to contribute to mycotoxin biosynthesis and pathogenicity [28,29,30,31,32]. Similarly, several peroxins such as MoPex5, MoPex6, MoPex7, MoPex13, MoPex14, MoPex17, MoPex19, and MoPex11 have been identified in *Magnaporthe oryzae* and demonstrated to be essential for plant infection [33,34,35,36,37]. In *Colletotrichum orbiculare*, the protein Fam1, which is similar to Pex22, has been found to be crucial for peroxisome formation and pathogenicity [38]. Additionally, a Pex22-like protein in *F. graminearum* has been characterized, highlighting its significant role in sexual and asexual reproduction, fatty acid utilization, deoxytrienol mycotoxin production, and pathogenicity [39]. However, the characterization of Pex22 in the rice blast fungus, an important model plant pathogenic fungus, remains unknown up to now. With the aim of gaining a better understanding of the role of the peroxin Pex22 in the pathogenesis of plant pathogenic fungi, particularly in appressorium-mediated plant infection, we characterized *MoPEX22* in *M. oryzae.* Our findings revealed that MoPex22 regulates the import of peroxisome matrix proteins by controlling the localization of MoPex4 and MoPex5, thereby influencing fatty acid metabolism, cell wall integrity, glycerol accumulation, and ultimately impacting the growth, functional appressorium formation, and pathogenicity of *M. oryzae*.

## 2. Materials and Methods

### 2.1. Strains and Culture Conditions

The wild-type strain used in this study was *M. oryzae* Guy11, as previously described [8,40,41,42]. *M. oryzae* Guy11 was employed for transformation, and all strains were cultured on complete medium (CM) plates at 28 °C. For the examination of colony morphology, Guy11 and the mutant strains were inoculated on the CM and SDC (rice straw decoction and corn medium) plates at 28 °C for 7 days, respectively. For conidiation, the strains were cultured on SDC plates in the dark at 28 °C for 7 days. Then, the aerial hyphae were stripped, and the remaining cultures were subjected to continuous illumination under fluorescent light for 3 days.

### 2.2. Gene Deletion and Complementation

A split marker strategy was used to replace *MoPEX22* with hygromycin phosphotransferase (*HPH*) in *M. oryzae* (Figure S2A) [43]. The allele was amplified with the primers 1F/2R, 3F/4R, HYGF/HYR, and YGF/HYGR (Table S1). After the transformation of wild-type Guy11 protoplasts, hygromycin-resistant transformants were screened using PCR with primers 5F/6R and confirmed using Southern blot (Figure S2B). For complementation assays, the *MoPEX22* gene containing a native promoter was amplified using PCR and cloned into a pYF11 plasmid to obtain the fusion plasmid using the yeast gap repair approach [44]. The constructed pYF11-*MoPEX22*-GFP was introduced into the protoplast of the ∆*Mopex22* mutant via transformation. Bleomycin-resistant transformants were isolated and examined under a fluorescence microscope (Leica DM5000B (Wetzlar, Germany), 100 × oil). The camera exposure is indicated in seconds (800 ms).

For observation of subcellular localization, a similar method was used; the fusion plasmids pYF11-RFP-*MoPEX4*, pYF11-*MoPEX5*-GFP, pYF11-*MoSEP3*-RFP, and pYF11-RFP-PTS1 were constructed and introduced into the protoplast of Guy11 or ∆*Mopex22* by transformation, respectively.

### 2.3. Virulence Assays and Infection Process Observation

Two-week-old rice seedlings (*Oryza sativa* cv. CO-39) were sprayed with a 5 mL conidial suspension of 5 × 10^4^ spores/mL containing 0.2% (*w*/*v*) gelatin and incubated at 28 °C in a moist and dark chamber for the first 24 h, followed by a 12 h/12 h light/dark cycle. Lesion formation was examined at 7 days after inoculation.

A total of 10 μL of a conidial suspension of 5 × 10^4^ spores/mL containing 0.2% (*w*/*v*) gelatin were dropped on wounded rice leaves and then incubated at 28 °C in a moist and dark chamber for the first 24 h, followed by a 12 h/12 h light/dark cycle. Lesion formation and size were assessed after 5 days of incubation.

To investigate the infection process, conidial suspension with a concentration of 5 × 10^4^ spores/mL in a 0.2% (*w*/*v*) gelatin solution was spotted on the lower epidermis of barley leaves and then incubated in a moist and dark chamber at 28 °C. After incubation with the spore suspensions for 24 h, four types (type 1, no penetration; type 2, only with a penetration peg or a single invasive hypha with no branch; type 3, invasive hyphae with one to three branches; and type 4, invasive hyphae with more than three branches) of invasive hyphae were observed in barley tissues.

### 2.4. Appressorium Turgor Assay

The appressorium turgor was measured using an incipient cytorrhysis assay and different concentrations of a glycerol solution or PEG8000. A total of 20 µL of a conidial suspension of 1 × 10^5^ spores/mL were dripped onto hydrophobic slides and incubated at 28 °C for 24 h. The water around the conidia was carefully removed and replaced with an equal volume of glycerol or PEG8000 at different concentrations. After 10 min, the collapsed appressoria was observed and recorded under a microscope.

### 2.5. Measurement of Lipid Mobilization

A 20 µL conidial suspension of 1 × 10^5^ spores/mL was dripped onto hydrophobic slides and incubated at 28 °C. The conidia and appressoria were stained using a Nile red solution [45] composed of a 50 mM Tris/maleate buffer (pH 7.5), 20 mg/mL polyvinylpyrrolidone, and 2.5 μg/mL Nile red oxazone (9-diethylamino-5H-benzo-a-phenoxazine-5-one, BBI, Shanghai, China). After 30 s of incubation, the lipid droplets in the conidia and appressoria were observed under the DM5000B fluorescence microscope (Leica, Wetzlar, Germany).

### 2.6. Assay for Fatty Acid Utilization

Strains were inoculated on a minimal medium (MM) and different variations of MM media at 28 °C for 7 days. The variations included replacing 1% (*w*/*v*) glucose with 50 mM NaAC, 1% (*v*/*v*) Tween 80, 1% olive oil, or 1% triolein. The colony diameter was measured and statistically analyzed.

### 2.7. Statistical Analysis

Error bars represent the standard deviation (SD), and values with asterisks represent significant differences between the mutant and both the wild-type and complemented strains (*, *p* < 0.05; **, *p* < 0.01). Representative data were analyzed using the one-way analysis of variance (ANOVA) using SPSS software (version 20.0; IBM Corporation New York City, NY, USA).

## 3. Results

### 3.1. MoPex22 Is Important for the Growth of M. oryzae

**Figure 1:**
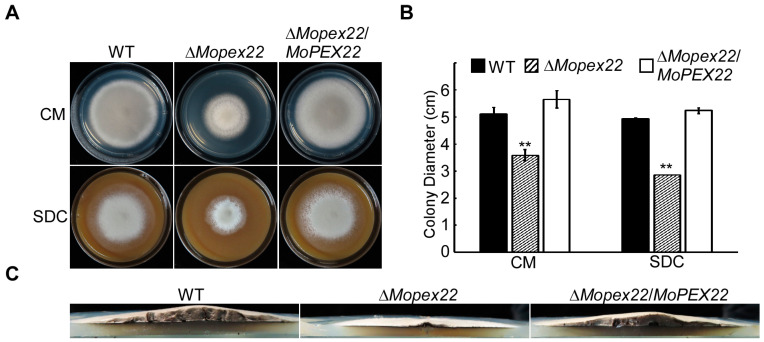
Vegetative growth of the ∆*Mopex22* mutant. (**A**) The indicated strains were inoculated on a complete medium (CM), straw decoction, and corn agar medium (SDC) and cultured at 28 °C for 7 days. (**B**) Statistical analysis of colony diameters of indicated strains. Error bars represent standard deviation (SD), and double asterisks indicate statically significant differences (**, *p* < 0.01). Means and standard deviations were calculated with data from three replicates. (**C**) Aerial hyphae growth is reduced in the ∆*Mopex22* mutant. Strains were grown under the same conditions as above, and colony-side views are displayed.

To identify the Pex22 protein in *M. oryzae*, a BLAST search of the *M. oryzae* genomic database was conducted (https://fungidb.org/fungidb/app/record/gene/MGG_02391, accessed on 10 March 2018) using the *S. cerevisiae* ScPex22 and *F. graminearum* FgPex22 protein sequences. One putative Pex22 protein, named MoPex22, was identified. Phylogenetic analysis revealed a high conservation of MoPex22, exhibiting a high amino acid sequence identity to Pex22 proteins found in other fungi (Figure S1). Additionally, domain prediction analysis showed the presence of an N-terminal TM domain in MoPex22, similar to other Pex22 proteins (Figure S1A). To examine the *MoPEX22* function in *M. oryzae*, the ∆*Mopex22* mutants and the complemented transformant ∆*Mopex22*/*MoPEX22* were generated (Figure S2). Then, the wild-type strain, ∆*Mopex22* mutant, and complemented strain were inoculated on CM and SDC plates at 28 °C for 7 days. Compared to the wild-type strain Guy11 and the complemented strain ∆*Mopex22*/*MoPEX22*, the ∆*Mopex22* mutant exhibited a noticeable growth defect with a significantly reduced colony size (Figure 1A,B). Furthermore, the ∆*Mopex22* mutant showed reduced aerial hyphae (Figure 1C). These findings suggest an important role of MoPex22 in the growth of *M. oryzae*.

### 3.2. MoPex22 Is Important for Appressorium-Mediated Penetration

**Figure 2:**
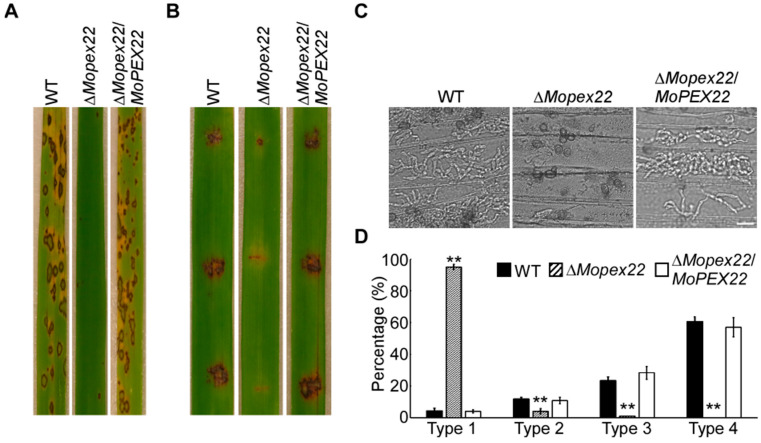
Virulence test of the ∆*Mopex22* mutant. (**A**) Rice (*Oryza sativa* cv. CO-39) seedlings were inoculated via conidial suspension. (**B**) Detached rice leaves wounded by abrasion were inoculated via conidial suspension. (**C**) Close observation of infection on barley. Excised barley leaves from 7-day-old barley seedlings were inoculated with conidial suspension. Infectious growth was observed at 24 h post-inoculation (hpi). Bar = 20 μm. (**D**) Statistical analysis for each type of infection hyphae. After incubation with the spore suspensions for 24 h, four types of invasive hyphae were observed in barley tissues. One hundred infecting hyphae (*n* = 100) were counted per replicate, and the experiment was repeated three times. Error bars represent standard deviation (SD), and double asterisks indicate statically significant differences (**, *p* < 0.01).

To investigate the role of MoPex22 in pathogenicity, conidial suspension of the wild-type strain Guy11, ∆*Mopex22* mutant, and complemented strain were sprayed onto rice seedlings, respectively. A large number of lesions appeared on the leaves of rice inoculated with the wild-type strain and complemented strain conidia. However, very few lesions were observed on the leaves of rice sprayed with mutant strain conidia (Figure 2A). The ∆*Mopex22* mutant caused minimal disease symptoms on wounded rice leaves compared to Guy11 and the complemented strain, both of which exhibited typical lesions (Figure 2B). To test the roles of MoPex22 during the infection process more extensively, we conducted infection assays on barley epidermal cells. Again, the penetration and invasive growth of the ∆*Mopex22* mutant were significantly attenuated. In the wild-type and the complemented strains, approximately 84% of the invasive hyphae were type 3 and type 4, with less than 16% being type 1 or type 2 hyphae. In contrast, the ∆*Mopex22* mutant showed less than 2% of type 3 and type 4 hyphae, while more than 98% were type 1 and type 2 hyphae (Figure 2C,D). These results indicate that MoPex22 is essential for normal appressorium-mediated penetration.

### 3.3. MoPex22 Regulates Appressorium Development

**Figure 3:**
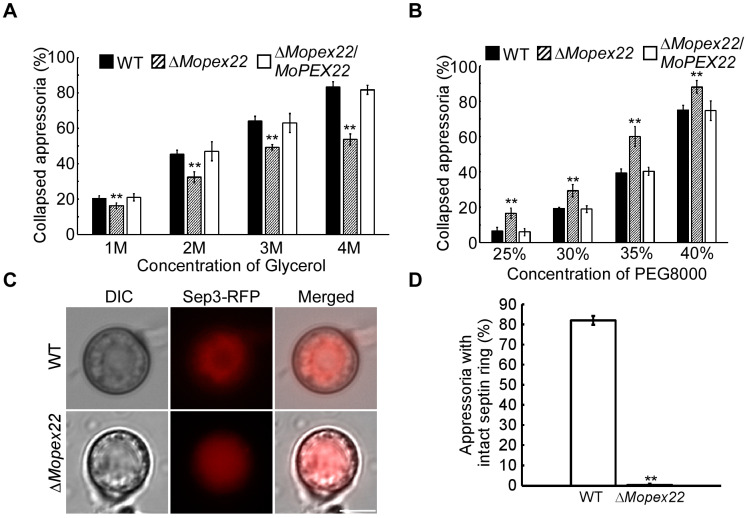
Functional appressorium formation of the ∆*Mopex22* mutant. (**A**,**B**) Cytorrhysis assay for appressorium turgor pressure. Drops of a conidial suspension (5 × 10^5^ conidia/mL) were placed on a hydrophobic surface and treated with different concentrations of glycerol or PEG8000. The percentage of the collapsed appressorium was calculated. One hundred appressoria (*n* = 100) were counted per replicate, and the experiment was repeated three times. Error bars represent standard deviation (SD), and double asterisks indicate statically significant differences (**, *p* < 0.01). (**C**) Septin morphologies in appressoria of WT and mutant. Bar = 5 μm. (**D**) Statistical analysis for septin ring in appressorium of WT and mutant. One hundred appressoria (*n* = 100) were counted per replicate, and the experiment was repeated three times. Error bars represent standard deviation (SD), and double asterisks indicate statically significant differences (**, *p* < 0.01).

To investigate the cause of the sharp decline in the appressorium-mediated host penetration in the ∆*Mopex22* mutant, we assessed the appressorium turgor pressure of the ∆*Mopex22* mutant by performing an incipient cytorrhysis assay. The collapse rates of the ∆*Mopex22* mutant appressorium treated with glycerol were significantly lower than those of the wild-type strain (Figure 3A). This phenomenon could be attributed to the defective cell wall of the ∆*Mopex22* mutant appressoria, which increased the permeability to glycerol. Therefore, polyethylene glycerol 8000 (PEG8000), which has a larger molecular size than glycerol, was used to treat the appressoria in the incipient cytorrhysis assay. The ∆*Mopex22* mutant appressoria were more prone to collapse compared to those of the wild-type strain (Figure 3B). These results suggest that the turgor in the ∆*Mopex22* mutant was reduced and that glycerol was able to traverse the cell wall of the ∆*Mopex22* mutant. In *M. oryzae*, the appressorium septin ring provides cortical rigidity and membrane curvature during pressure production, which is necessary for the protrusion of a rigid penetration peg to breach the host cuticle [7,8,46]. Considering that the ∆*Mopex22* mutant formed a functionally defective appressorium with reduced penetrability, we examined the organization of one core septin, MoSep3-GFP, in the ∆*Mopex22* mutant. A clear septin ring was visible surrounding the appressorium pore after induction for 24 h on the hydrophobic surface in the WT. However, in nearly all of the appressoria of the ∆*Mopex22* mutant, condensed RFP ball-like structures were observed instead of regular septin rings (Figure 3C,D). These results indicate that MoPex22 is involved in regulating the development of functional appressorium in *M. oryzae*.

### 3.4. MoPex22 Is Involved in Lipid Metabolism in M. oryzae

**Figure 4:**
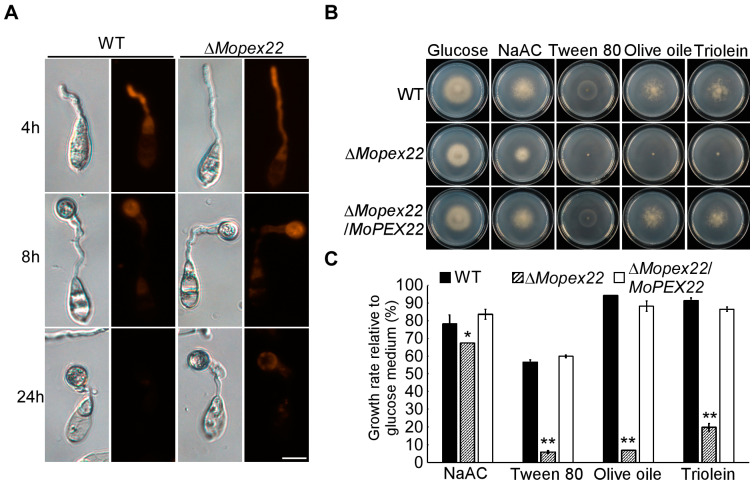
Lipid metabolism of the ∆*Mopex22* mutant. (**A**) Deletion of *MoPEX22* delayed lipid mobilization and degradation during appressorium development. Conidia of the wild-type strain Guy11 and mutant were incubated on a hydrophobic membrane and allowed to form appressoria. Samples at different time points were stained with Nile red and examined microscopically. Bar = 10 μm. (**B**,**C**) Lipid utilization via Δ*Mopex22* and the wild-type strain Guy11. The strains were cultured on a minimal medium with glucose (1%), NaAc (50mM), Tween 80 (1%), olive oil (1%), or triolein (1%) as the sole carbon source for 7 days. The colony diameter was measured and statistically analyzed. Error bars represent standard deviation (SD), and asterisks indicate statically significant differences (*, *p* < 0.05, **, *p* < 0.01). Means and standard deviations were calculated with data from three replicates.

To investigate the role of MoPex22 in lipid mobilization, Nile red staining was performed during appressorium development. In the Guy11 strain, lipid bodies in conidia were transported to the appressorium and underwent degradation. By 24 h, there were nearly no liposomes in the wild type (Figure 4A). However, in the ∆*Mopex22* mutant, the transport and degradation of lipid bodies in conidia were delayed during appressorium development. The lipid bodies remained detectable in the appressoria and even in the conidia at 24 h (Figure 4A). These findings indicate that MoPex22 regulates lipid mobilization during appressorium development. Furthermore, the mutant exhibited limited growth in media containing fatty acids such as Tween 80, olive oil, and triolein as the sole carbon source, providing evidence for the involvement of MoPex22 in fatty acid degradation. Additionally, despite the mutant’s capability to utilize NaAc, which represents both the product of fatty acid oxidation and the initiator of the glyoxylate cycle, its growth rate relative to that of the MM medium also decreased. Therefore, MoPex22 plays a crucial role in fatty acid degradation and has an impact on subsequent biochemical processes like the glyoxylate cycle.

### 3.5. Deletion of MoPEX22 Reduced the Tolerance of the Mutant to SDS, CR, and H_2_O_2_

**Figure 5:**
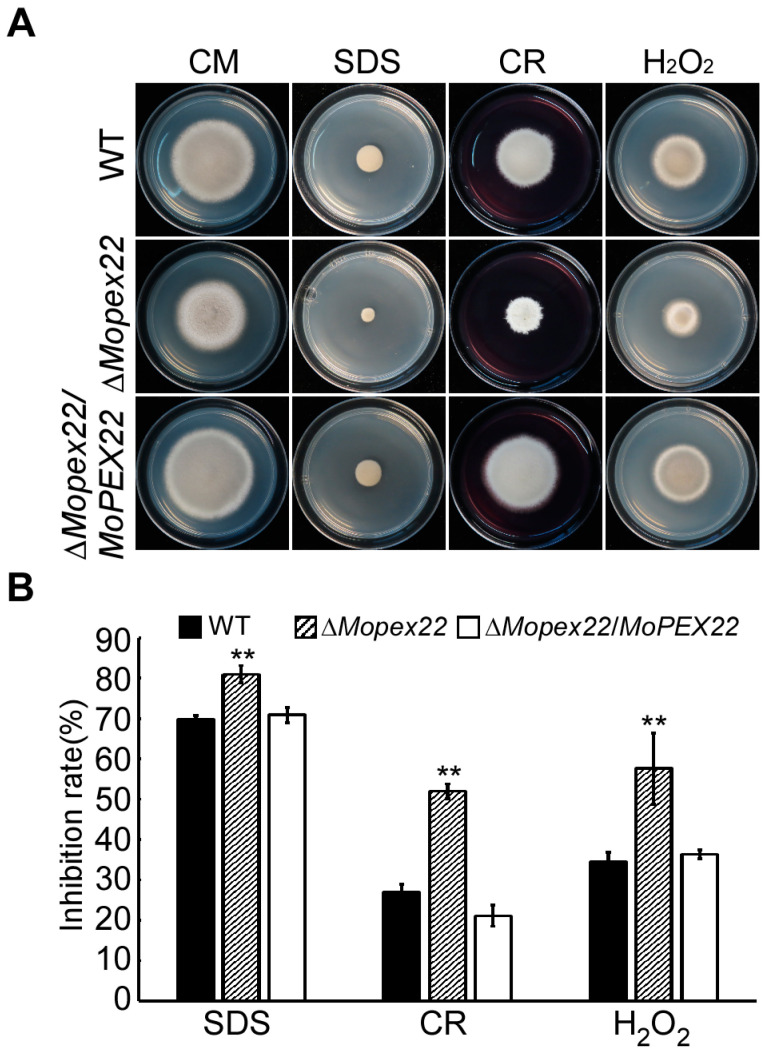
Tolerance test of the ∆*Mopex22* mutant to SDS, CR, and H_2_O_2_. (**A**) The wild-type strain and the Δ*Mopex22* strain were inoculated on a CM medium with or without SDS (0.01% *w*/*v*), CR (400 mg/mL), or H_2_O_2_ (2.5 mM), and cultured at 28 °C for 7 days. (**B**) The colony diameters were measured, and the inhibition rate was statistically analyzed. Inhibition rate (%) = (diameter of untreated strain − diameter of treated strain)/(diameter of untreated strain) × 100%. Error bars represent standard deviation (SD), and double asterisks indicate statically significant differences (**, *p* < 0.01). Means and standard deviations were calculated with data from three replicates.

Peroxisomal metabolism is intricately linked to cell wall biogenesis and fungal pathogenicity. To investigate the impact of *MoPEX22* deletion on cell wall integrity in *M. oryzae*, the Δ*Mopex22* mutant was inoculated on CM supplemented with Sodium dodecyl sulfate (SDS) or Congo red (CR), two agents known to disrupt cell wall synthesis. The mutant exhibited increased sensitivity to both agents compared to the wild type (Figure 5), suggesting that MoPex22 was involved in maintaining the cell wall integrity. Another vital metabolic function of peroxisomes is the elimination of reactive oxygen species (ROS), which is essential for fungal infection. To evaluate the mutant’s ability to degrade ROS, the growth on CM supplemented with H_2_O_2_ was compared. The Δ*Mopex22* strain displayed significantly reduced tolerance to H_2_O_2_ (Figure 5), providing evidence for the participation of MoPex22 in *M. oryzae*’s antioxidant capacity.

### 3.6. MoPex22 Localizes to Peroxisomes and Regulates the Import of Matrix Proteins into Peroxisomes

**Figure 6:**
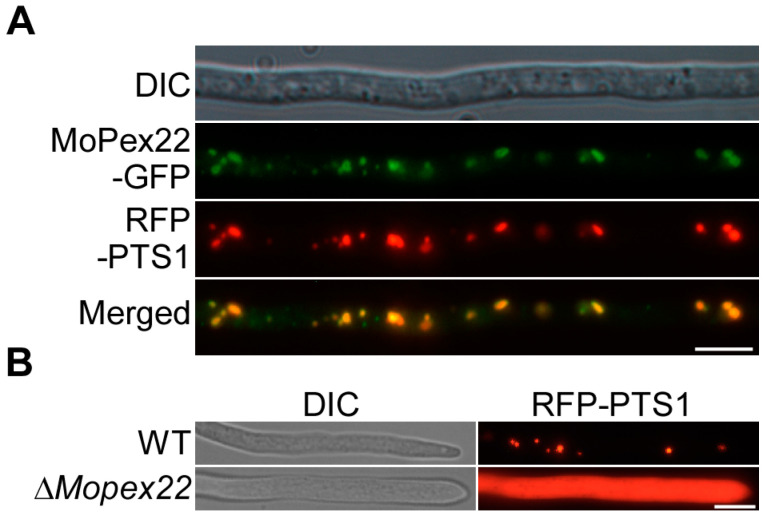
Subcellular localization of RFP-PTS1 in the ∆*Mopex22* mutant. (**A**) MoPex22 is located in the peroxisome. Hyphae of the transformants expressing the *MoPEX22*-GFP and RFP-PTS1 were observed after incubation in liquid CM for 48 h. Bar = 10 μm. (**B**) Observation of RFP-PTS1 in hyphae of a wild type and the ∆*Mopex22* mutant. Hypha of the WT and mutants expressing RFP-PTS1 were cultured in a liquid complete medium for 48 h and were observed. Bar = 10 μm.

To investigate the subcellular localization of MoPex22, the strain expressing *MoPEX22*-GFP and the peroxisomal marker RFP-PTS1 were constructed and examined using fluorescence microscopy. The MoPex22-GFP displayed fluorescence in a punctate pattern that frequently overlapped with the red fluorescence of the peroxisomal marker RFP-PTS1, confirming its localization in peroxisomes (Figure 6A). To explore the role of MoPex22 in peroxisomal function, RFP-PTS1 was introduced into the wild-type strain and ∆*Mopex22* mutant. In the wild-type strain, the RFP signals were abundant punctate and localized to peroxisomes. However, in the Δ*Mopex22* mutant, the RFP signals appeared to diffuse in the cytoplasm, with only a few localized spots (Figure 6B). This observation suggests that the deletion of *MoPEX22* impairs the import of peroxisomal matrix proteins into peroxisomes. Therefore, MoPex22 plays an essential role in peroxisome biogenesis in *M. oryzae*.

### 3.7. MoPex22 Anchors MoPex4 to Peroxisomes

**Figure 7:**
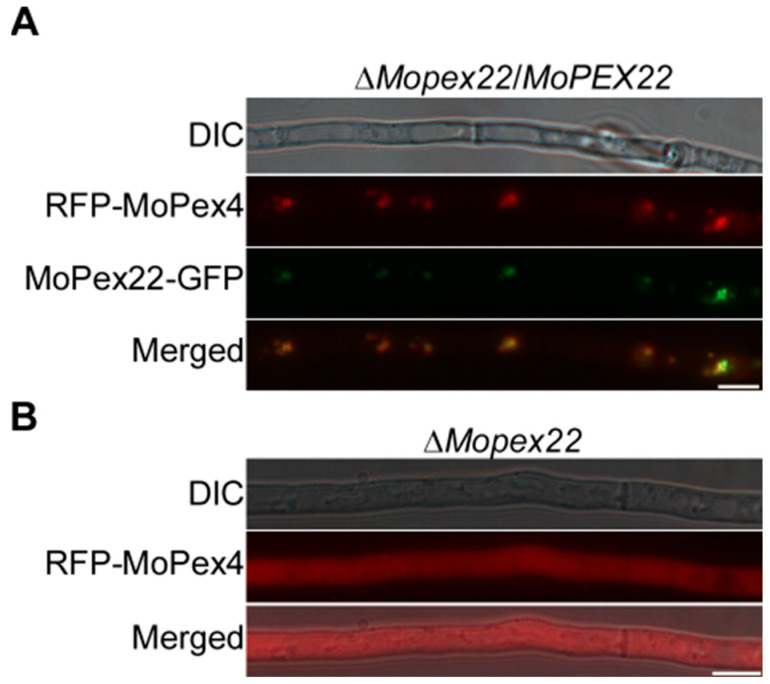
Subcellular localization of MoPex4 in the ∆*Mopex22* mutant. (**A**) MoPex22 colocalizes with MoPex4 in peroxisomes in hyphae. Vegetative hyphae of the transformants expressing the *MoPEX22*-GFP and RFP-*MoPEX4* were observed after incubation in liquid CM for 48 h. Bar = 10 μm. (**B**) MoPex22 affects the subcellular localization of MoPex4. Vegetative hyphae of the ∆*Mopex22* expressing the RFP-*MoPEX4* were observed after incubation in liquid CM for 48 h. Bar = 10 μm.

To investigate the impact of *MoPEX22* deletion on MoPex4, the RFP-*MoPEX4* plasmid was constructed and introduced into ∆*Mopex22*/*MoPex22* strain and ∆*Mopex22* strain. The RFP-*MoPEX4* displayed punctate fluorescence patterns that frequently overlapped with *MoPEX22*-GFP, indicating the co-localization of MoPex4 and MoPex22 within peroxisomes (Figure 7A). However, in the ∆*Mopex22* mutant, RFP-MoPex4 was dispersed throughout the cytoplasm, suggesting that MoPex4 was not anchored to the peroxisome due to the deletion of *MoPEX22* (Figure 7B). These results suggest that MoPex22 plays a crucial role in anchoring MoPex4 to peroxisomes.

### 3.8. MoPex22 Regulates the Localization of MoPex5

**Figure 8:**
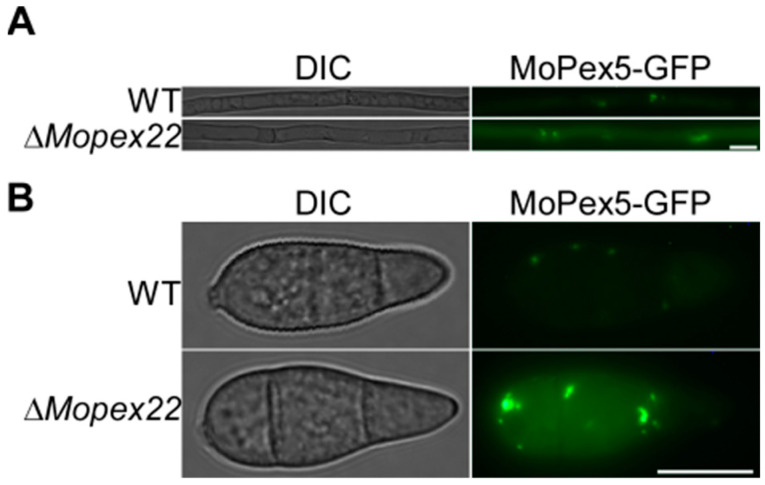
Subcellular localization of MoPex5 in the ∆*Mopex22* mutant. (**A**,**B**) Subcellular localization of MoPex5-GFP in hyphae and conidia of WT and ∆*Mopex22* strains. Vegetative hyphae of the WT and ∆*Mopex22* expressing the *MoPEX5*-GFP were observed after incubation in liquid CM for 48 h. Bar = 10 μm.

To investigate the effect of *MoPEX22* deletion on MoPex5, which is a PTS receptor, the *MoPEX5*-GFP construct was generated and introduced into the wild-type strain Guy11 and ∆*Mopex22* mutant via transformation, respectively. The transformants expressing the *MoPEX5*-GFP construct were examined using fluorescence microscopy. The Δ*Mopex22* mutant exhibited a stronger fluorescence signal compared to the wild-type strain, particularly in the punctate peroxisomes. Notably, this difference was more pronounced in conidia (Figure 8A,B). These findings suggest that the absence of *MoPEX22* leads to abnormal localization of MoPex5.

## 4. Discussion

During infection, *M. oryzae* can form a specialized structure called appressorium to penetrate host cells [2]. The formation and development of the appressorium involve several cellular processes, including cell wall thickening, melanin layer formation, glycerol accumulation to generate sufficient turgor pressure, cytoskeletal rearrangement to form mechanical forces and penetration pegs [7,8,47,48]. In this study, we characterized the peroxin-coding gene *MoPEX22* in *M. oryzae*. Our data showed that the Δ*Mopex22* mutant was defective in growth and appressorium development and exhibited reduced pathogenicity. We found that the appressoria of the Δ*Mopex22* mutant were defective in penetrating plant cells. Moreover, we subsequently revealed the roles of MoPex22 in coordinating peroxisome matrix transport, lipid utilization, septin reassembly, oxidative response, and cell wall integrity, which are important for functional appressorium formation and host penetration. To our knowledge, this is the first report of a *PEX22* homolog playing an important role in *M. oryzae*.

Peroxisomes play a crucial role in various developmental processes in fungi. The transport of matrix proteins directly affects peroxisome function [49]. In *S. cerevisiae*, Pex22 participates in receptor recycling by anchoring the ubiquitin-conjugating enzyme Pex4 to the cytosolic face of the peroxisomal membrane [24]. In this study, MoPex22 localizes to peroxisomes in *M. oryzae*, aligning with findings in other organisms. The wild-type strain expressing RFP-PTS1 exhibited abundant punctate organelles with visible RFP signals, which localized to peroxisomes. However, in the Δ*Mopex22* mutant, RFP-PTS1 labeling dispersed throughout the fungal cytosol, indicating the impaired import of peroxisomal matrix proteins into peroxisomes due to the *MoPEX22* mutation. In this study, MoPex4 and MoPex22 colocalize in peroxisomes. After deleting *MoPEX22*, MoPex4 dispersed in the cytoplasm. Moreover, in the Δ*Mopex22* mutant, the MoPex5-GFP became more concentrated in peroxisomes, while abnormal expression was observed in the cytoplasm. In *S. cerevisiae*, the Pex5 receptor undergoes recycling during peroxisomal matrix protein transport. The Pex4 monoubiquitination of Pex5 promotes the release of Pex5 from the peroxisomal membrane [50]. Pex4 cannot function independently, and it requires Pex22 to anchor it to the peroxisomal membrane for normal activity [24,51]. In the ∆*Mopex22* mutant, MoPex4 fails to localize to peroxisomes, leading to impaired recycling of MoPex5 receptors. This ultimately affects the transport of peroxisomal matrix proteins. Therefore, MoPex22 regulates the peroxisomal localization of MoPex4, the recycling of MoPex5 receptors, and overall peroxisome function in *M. oryzae*.

Impaired peroxisomal matrix protein transport has a significant impact on peroxisome function, which is crucial for fungal virulence [49]. The ∆*Mopex22* mutant conidia can germinate and form appressoria, but their ability to penetrate host cells is significantly reduced. Tests with different concentrations of glycerol and PEG8000 revealed that the appressoria of the Δ*Mopex22* mutant lacked adequate turgor pressure due to reduced glycerol accumulation and defects in the appressorial cell wall. The lipid droplet metabolism within the peroxisomes plays a crucial role in producing glycerol necessary for achieving the required turgor pressure [47]. Abnormal lipid degradation during spore germination and appressorium formation in the ∆*Mopex22* mutant hinders the accumulation of adequate glycerol. In *M. oryzae*, deletion of *MoPEX6* also blocked the fatty acid β-oxidation, resulted in enlarged lipid droplets, and rendered appressoria non-infectious against rice [5,52]. In *C. orbiculare*, the ∆*pex13* mutant forms appressoria, lacking infectivity due to blockage of the fatty acid β-oxidation [53].

The turgor threshold requires lipid droplet metabolism for glycerol production, as well as the thickening of the cell wall and the formation of the melanin layer to prevent glycerol leakage [7,47]. In this study, the collapse rates of the ∆*Mopex22* mutant appressorium treated with glycerol were significantly lower than those of the wild-type strain. The ∆*Mopex22* mutant appressoria were more prone to collapse compared to those of the wild-type strain in the incipient cytorrhysis assay with PEG8000. This phenomenon could be attributed to the defective cell wall of the ∆*Mopex22* mutant appressoria, which increased glycerol permeability. The intermediates generated via β-oxidation and the glyoxylate cycle in peroxisomes serve as prerequisite materials for cell wall components [54,55]. Acetyl-CoA, produced via fatty acid b-oxidation in the peroxisome, acts as a precursor for melanin synthesis [33,56,57,58]. ∆*Mopex22* exhibits slow growth on media with fatty acids of varying chain lengths as the sole carbon source, supporting the role of MoPex22 in regulating fatty acid β-oxidation and the glyoxylate cycle. Impaired β-oxidation and glyoxylic acid cycling may result in defective cell wall synthesis and abnormalities in the cell wall structure. β-oxidation and the glyoxylate cycle occur in the peroxisome, where the associated enzymes are transported to function [49,59]. The defective transport of peroxisomal proteins caused by *PEX22* deficiency may hamper enzyme transportation and lead to cell wall defects. In *M. oryzae*, the loss of *PTH2,* which encodes carnitine acyltransferase, results in impaired acetyl-coA transport, compromised cell wall synthesis, and reduced appressorium penetration into host cells [54]. Additionally, the ∆*Mopex22* strain exhibited increased sensitivity to cell wall inhibitors such as Congo red and SDS. Therefore, the deletion of *MoPEX22* resulted in cell wall damage, heightened sensitivity to external stress, and reduced turgor pressure.

Appressorium-mediated host penetration necessitates not only sufficient turgor pressure but also the directed transmission of turgor pressure through the penetration peg into host cells [7,8]. The septins at the base of the appressorium provide cortical rigidity in the initially wall-less region and convert isotropic expansion into a mechanical force directed toward the host cell [8]. Disruption or loss of septin ring assembly eliminates appressorium-mediated host penetration and invasive hyphal growth in *M. oryzae* [46,60,61]. Septin rings were rarely found in the ∆*Mopex22* mutant, resulting in further defects in the formation of invasive pegs. We speculate that the failure to assemble the septin ring is a synergistic effect caused by insufficient turgor pressure in the appressorium and defects in cell wall integrity. After all, when the isotropic expansion of the pressurized appressorium reaches a specific threshold, Sln1 activates the Nox2-NoxR NADPH oxidase through the Pkc1-dependent cell-integrity pathway. This activation recruits septins to the appressorium pore and reorganizes F-actin to facilitate force generation and polarized growth [7].

Peroxisomes contain various enzymes involved in the oxidative stress response, such as catalase and superoxide dismutase [62]. ROS-scavenging enzymes, mainly catalase and peroxidase, with the PTS1 signal sequence, are found in peroxisomes [49,63]. In this study, the Δ*Mopex22* mutant exhibited significantly reduced tolerance to H_2_O_2_. The RFP-PTS1 labeling dispersed throughout the fungal cytosol, indicating impaired import of peroxisomal matrix proteins into peroxisomes in the Δ*Mopex22* mutant. MoPex22 may affect the response of rice blast fungus to oxidative stress by influencing the transport of ROS-scavenging enzymes between the cytoplasm and peroxisomes. Deletion of *PEX13*, *PEX14*, and *PEX33* in *F. graminearum* and *PEX13* and *PEX14* in *M. oryzae* also showed increased sensitivity to ROS [29,64].

In summary, this study reveals that MoPex22 regulates the peroxisomal localization of MoPex4 and the recycling of MoPex5 receptors, thereby controlling the transport of peroxisome matrix proteins in the rice blast fungus. It also participates in fatty acid b-oxidation, turgor formation in the appressorium, regulation of cell wall integrity, and oxidative response. These regulatory functions ultimately govern the growth, appressorium development, and pathogenicity of the rice blast fungus. These findings provide valuable insights into the roles of Pex22 and peroxisomes in the pathogenic process of fungi.

## Data Availability

Data are contained within the article and supplementary materials.

## Supplementary Materials

The following supporting information can be downloaded at https://www.mdpi.com/article/10.3390/jof10020143/s1, Figure S1: Phylogenetic tree and structure analysis of Pex22 from selected fungi. (A) Analysis of domain in Pex22 protein from selected fungi. The domain was analyzed using the Simple Modular Architecture Research Tool (SMART). All Pex22 proteins were downloaded from the NCBI database, and their accession numbers are as following: *M. oryzae* (*Magnaporthe oryzae* XP_003709168.1), *C. truncatum* (*Colletotrichum truncatum* XP_036575598.1), *R. terebratum* (*Rostrohypoxylon terebratum* KAI1087461.1), *F. graminearum* (*Fusarium graminearum* XP_011317390.1), *N. crassa* (*Neurospora crassa* XP_955773.2), *A. fumigatus* (*Aspergillus fumigatus* KAF4274661.1), *A. nidulans* (*Aspergillus nidulans* XP_658581.1), *S. cerevisiae* (*Saccharomyces cerevisiae* NP_009346.1). (B) Phylogenetic tree of Pex22 protein from selected fungi. Sequence alignments were performed using the Clustal_W program, and the nighbor-joining tree was constructed with 1000 bootstrap replicates by the use of MEGA 7.0.; Figure S2: Targeted gene replacement of *MoPEX22*. (A) Schematic diagram of *MoPEX22* gene knockout pattern diagram. HY and YG indicate parts of the hygromycin B phosphotransferase gene cassette. The split HYG templates for *MoPEX22* disruption were amplified and ligated to the specific flanking sequences by overlap-PCR. (B) Southern blot confirmation of the *MoPEX22* gene deletion mutants. Southern blots of *Pst* I-digested genomic DNA of the wild-type, and Δ*Mopex22* mutant strains hybridized with the *MoPEX22* and *HPH* probes, respectively; Table S1: Primers used in this study.
