# Supplementary material for: Peroxin MoPex22 Regulates the Import of Peroxisomal Matrix Proteins and Appressorium-Mediated Plant Infection in Magnaporthe oryzae
Source: J Fungi (Basel). 2024 Feb 10;10(2):143. doi: 10.3390/jof10020143 (PMC10890347; doi:10.3390/jof10020143)
Supplement: Supplementary file 1 [file jof-10-00143-s001.zip › Table S1.pdf]

Table S1 Primers used in this study.

Primer	Sequence (5’-3’)	Application
MGG02391/1F	GCAGGGAAGGACGATGAAGTT	amplify *MoPEX22* 5’ flank sequence
MGG02391/2R	TTGACCTCCACTAGCTCCAGCCAAGCCGCGGAGGTTTGTCAAGATGG	amplify *MoPEX22* 5’ flank sequence
MGG02391/3F	CAAAGGAATAGAGTAGATGCCGACCGGGATGCAGGGAAACGGTATG	amplify *MoPEX22* 3’ flank sequence
MGG02391/4R	CCTGGTCGGTGCTGGTTATG	amplify *MoPEX22* 3’ flank sequence
HYGF	GGCTTGGCTGGAGCTAGTGGAGGTCAA	amplify *HPH* N-terminal sequence
HYR	TATTGACCGATTCCTTGCGGTCCGAA	amplify *HPH* N-terminal sequence
YGF	GATGTAGGAGGGCGTGGATATGTCCT	amplify *HPH* C-terminal sequence
HYGR	CGGTCGGCATCTACTCTATTCCTTTG	amplify *HPH* C-terminal sequence
MGG02391/5F	AGACGGTTGCGGCTGGTGT	amplify *MoPEX22* probe sequence
MGG02391/6R	CGAGGGCTTGGCTGTAGATTGT	amplify *MoPEX22* probe sequence
MGG02391comF	TATAGGGCGAATTGGGTACTCAAATTGGTTAGCCTTCGTTGTCATCTGTTATGTC	*MoPEX22* complementation
MGG02391comR	CCCGGTGAACAGCTCCTCGCCCTTGCTCACCTCCCTACCCTGAACCCTCCT	*MoPEX22* complementation
MGGRFP00180/F	CGCGCCGAGGGCCGCCACTCCACCGGCGCCGCGCCCAAATCCGACCGGT	Construction of RFP-*MoPEX4*
MGGRFP00180/R	TTACTTGTACAGCTCGTCCATGCCGAGAGTTCAAGG	Construction of RFP-

	CCCATCATATCTCCCACC	*MoPEX4*
MGG10840GFP/F	TATAGGGCGAATTGGGTACTCAAATTGGTTTGTTAG AGCAACCCAATGGTATTTT	Construction of *MoPEX5*-GFP
MGG10840GFP/R	CCCGGTGAACAGCTCCTCGCCCTTGCTCACAAAGT CAAAGTCCTTCCTGAAGACAT	Construction of *MoPEX5*-GFP
MGG01521RFP/F1	TATAGGGCGAATTGGGTACTCAAATTGGTTGGTGCA TCGGCCCCACTATTAG	Construction of *MoSEP3*-RFP
MGG01521RFP/R1	GAACTCCTTGATGACGTCCTCGGAGGAGGCACGGA GTGAGAAACCCTTCCTCTTG	Construction of *MoSEP3*-RFP
RFP-PTS1/F	TTTCGTAGGAACCCAATCTTCAAAATGGCCTCCTCC GAGGACGTCATCA	Construction of RFP-PTS1
RFP-PTS1/R	TTACTTGTACAGCTCGTCCATGCCGAGAGTTTACAG CTTCGACAGGAACAGGTGGTGGCGGCCCT	Construction of RFP-PTS1
RFP/F1	TTTCGTAGGAACCCAATCTTCAAAATGGCCTCCTCC GAGGACGTCATC	Construction of RFP-PTS1
RFP/R1	GGCGCCGGTGGAGTGGCGGC	Construction of RFP-PTS1
